# Retraction Note: Marangoni convection in dissipative flow of nanofluid through porous space

**DOI:** 10.1038/s41598-023-49474-7

**Published:** 2023-12-12

**Authors:** Ikram Ullah, Mohammad Mahtab Alam, Muhammad Irfan Shah, Wajaree Weera

**Affiliations:** 1 Department of Natural Sciences and Humanities, University of Engineering and Technology, Mardan, 23200 Pakistan; 2 Department of Basic Medical Sciences, College of Applied Medical Science, King Khalid University, 61421 Abha, Saudi Arabia; 3 Department of Sciences and Humanities, National University of Computer and Emerging Sciences, Islamabad, 44000 Pakistan; 4 Department of Mathematics, Faculty of Science, Khon Kaen University, Khon Kaen, 40002 Thailand

Retraction of: *Scientific Reports* 10.1038/s41598-023-30795-6, published online 18 April 2023

The Editors have retracted this Article. After being informed of concerns regarding the content of the Article, the Authors were requested to provide the original raw data underlying this study, but despite repeated requests were unable to provide this. In addition, the Editors have developed concerns regarding the veracity of the authorship of this Article. The Editors have therefore lost confidence in the reliability and the integrity of the work presented in this Article.

All Authors disagree with the retraction.

